# Australian Podiatry Research in Workforce and Education: A Bibliometric Analysis

**DOI:** 10.1002/jfa2.70168

**Published:** 2026-06-02

**Authors:** Malia Ho, Peta Tehan, Matthew R. Carroll, Cylie Williams

**Affiliations:** ^1^ School of Primary and Allied Healthcare Monash University Victoria Australia; ^2^ School of Clinical Sciences Monash University Victoria Australia; ^3^ School of Allied Health Auckland University of Technology Auckland New Zealand

**Keywords:** bibliometric analysis, education, foot, podiatry, publications, research, workforce

## Abstract

**Background:**

To conduct a bibliographic analysis of English language research pertaining to podiatry workforce and education by Australian authors.

**Methods:**

The Scopus database search was conducted to identify all Australian workforce and education‐related articles published by podiatric authors in English from 1970 to 2024. Bibliometric analysis was performed using Biblioshiny, a web‐based graphical interface for the bibliometric R package. Citations, journals, authors, institutions, and countries were described. Publications were manually categorised according to research type, level of evidence and funding source.

**Results:**

The search strategy yielded 105 eligible articles, which received a total of 975 citations and were published by 338 authors in 33 journals. The most frequent journal was *Journal of Foot and Ankle Research* (34 articles; 32%), and the most frequently cited was the University of South Australia (affiliation of 85 authors). Most Australian workforce and education articles published by podiatrists focused on health and social care services research (*n* = 60; 57%) and only five articles (5%) provided level I evidence. Fifty‐six articles (53%) reported no research funding. Research generally fell under four themes.

**Conclusion:**

Workforce and education research make up a small percentage of podiatry‐related research. Most are published in low‐impact journals with low citations. The articles are highly collaborative, with multiple authors nationally and internationally. Studies are driven by academics with a vested interest in workforce and education issues, with little or no funding. There is a lack of research in continuing professional development educational activities and workforce in the private sector. Being a small profession, podiatry data may be missed in large allied health workforce studies. Podiatry workforce and education research must be driven by the profession, thereby creating opportunities for sustainable podiatry career growth.

## 1 Background

According to workforce data from the Podiatry Board of Australia Annual Report 2024/2025, there are 6210 registered podiatrists, which is an increase of 1.2% compared to the previous year. A total of 303 (5%) hold endorsement for scheduled medicines. Just over half, 3666 (59%) identify as female, and 36% are within the 25–34 age range. A total of 0.7% identify as Aboriginal or Torres Strait Islander [1]. There are nine universities offering podiatry entry‐level qualifications in Australia. Seven universities offer undergraduate education and training (Central Queensland University, Charles Sturt University, La Trobe University, The University of Newcastle, Queensland University of Technology, Adelaide University, Western Sydney University) and two universities offer this at a post‐graduate level (Monash University, University of Western Australia).

Podiatry education and training equip students with current practical and critical thinking skills to meet the ever‐evolving industry needs. Although all podiatry programs are accredited by the Podiatry Accreditation Committee of the Podiatry Board, the accreditation exercise ensures that the graduates meet the threshold requirements of the Podiatry Professional Standards. Research in podiatry education can engage consumers and stakeholders to evaluate teaching methods and new program delivery models to improve podiatry pedagogy [2]. This ensures that new graduates are industry‐ready and are able to provide treatments that are clinically and culturally safe.

Research in the podiatry workforce is vital to inform workforce planning, including developing strategies to ensure sufficient trained podiatrists to meet consumer demands [3]. For example, research is necessary to ensure that podiatry remains an attractive career option [4, 5, 6], and strategies can be put in place to retain podiatrists in the workforce and reduce attrition [7]. This is probably most relevant for clinicians working in rural and remote areas [8].

Research into podiatry health service provision is critical to ensure evidence‐based practice, to improve health outcomes, and reduce healthcare costs [9]. Research in this area helps decision makers create policies that are relevant, support the development of specialist career pathways, and ensures that healthcare services are most efficiently utilised [10].

To date, there are no studies that have investigated the profile of research studies focussing on the workforce and education in podiatry in Australia. Therefore, as part of a national research priorities initiative, this study set out to map the landscape of the Australian workforce and education research through a comprehensive bibliographic analysis. Our objective was to identify who is driving this research, the nature and location of the work being undertaken, and the sources of funding supporting the podiatric workforce and education in Australia.

## 2 Method

We conducted a bibliometric analysis of articles between January 1970 and December 2024 using data from the Scopus database (Elsevier, Amsterdam, the Netherlands). The Scopus database was chosen due to its broader journal coverage compared to PubMed and the Web of Science [11, 12]. The study is reported using the bibliometric analysis guidelines suggested by Montazeri et al. [13]. The detailed methodology is reported in the full study, as this is a sub‐study utilising workforce and education‐related research [14].

### 2.1 Search Strategy

The search strategy included three concepts: (i) workforce retention and satisfaction, (ii) professional development (iii) student supervision and education. Full details are provided in Supporting Information S1.

### 2.2 Article Selection

The titles and abstracts of all articles were downloaded from the Scopus database and exported into a systematic review application (Covidence, Veritas Health Innovation, Melbourne, Australia; P.T., M.R.C.). Titles, abstracts, and full‐text articles were then independently screened by two researchers (M.H., C.W.) with disagreements resolved by discussion or a third researcher (P.T.). Eligible publications were original studies or systematic reviews published in English, completed at an Australian education or healthcare institution, in an Australian cohort of participants, where at least one author had an Australian affiliation. For systematic reviews, the first or last author was required to have had an Australian affiliation. Eligible research was deemed to be relevant if it demonstrated applicability to podiatry practice, specifically, if its findings related to education that could inform clinical decision‐making or patient care, workforce planning, and research on ‘how’ to educate podiatry students or in the post‐graduate education setting. Laboratory‐based or pre‐clinical studies were not included because they were not deemed directly relevant to podiatry clinical practice. Guidelines, consensus documents, case studies, research letters, editorials, commentaries and conference abstracts were not included.

### 2.3 Data Extraction

Articles were imported into Biblioshiny (*bibliometrix* package version 4.1.4; University of Naples Federico II, Naples, Italy) [15]. The following characteristics were extracted from each article: year of publication, journal name, 2023 Impact Factor (using Journal Citation Reports [Clarivate Analytics, Philadelphia, Pennsylvania, USA]), number of citations (as recorded in the Scopus database [Elsevier, Amsterdam, Netherlands]), author names, and institutional and country affiliation.

### 2.4 Data Synthesis

Research type was categorised according to the United Kingdom Clinical Research Collaboration (UKCRC) Health Research Classification System [16]. This system classifies types of research activities using 48 codes within eight groups: (i) underpinning research, (ii) aetiology, (iii) prevention of diseases and conditions and promotion of well‐being, (iv) detection, screening and diagnosis, (v) development of treatments and therapeutic interventions, (vi) evaluation of treatments and therapeutic interventions, (vii) management of diseases and conditions, and (viii) health and social services research. Level of evidence was manually assigned to each study using the National Health and Medical Research Council (NHMRC) criteria [16], which specifies the following: (i) level I: evidence from a systematic review of all relevant randomised controlled trials; (ii) level II: evidence from at least one properly designed randomised controlled trial; (iii) level III: evidence from other well‐designed experimental or analytical studies; (iv) level IV: evidence from descriptive studies. Funding sources were documented according to the Australian Government Higher Education Research Data Collection (HERDC) specifications [17] as follows: (i) Category 1: Australian competitive grant research and development income; (ii) Category 2: other public sector research and development income; (iii) Category 3: industry and other research and development income; (iv) Category 4: cooperative research centre research and development income.

We also undertook a content framework analysis [18] of article titles to understand the different types of research. We used the principles of content framework analysis to guide this approach. In this analysis, one researcher (C.W.) read through all titles and formed topic themes. This took an inductive approach where as a new theme was identified, all prior data were recoded. When all were coded, the themes were checked with the full author team and coded by a second researcher (M.H.) with disagreements in alignment between title and theme by a third researcher (P.T.). Themes were then displayed in a tree map created with Excel (Version 16, Microsoft, 2026). Where articles overlapped, we used a best‐fit approach agreed on by two researchers (M.H., C.W. or P.T.).

### 2.5 Article Characteristics

The search strategy yielded 105 eligible articles (see Supporting Information S2). Table 1 presents the characteristics of these articles, noting that articles in workforce and education received a total of 975 citations and were published by 338 authors. Table 2 shows the characteristics of the top 10 most frequent journals in which these articles were published and the respective number of publications per journal. The top journal publishing articles in workforce and education was the *Journal of Foot & Ankle Research*.

**TABLE 1:** Publication characteristics.

	Workforce/education
Years articles published in	1998–2024
Total number of articles	105
Original articles	99
Systematic reviews	6
Mean years from publication (SD)	6.72 (5.72)
Mean citations per year per article (SD)	9.3 (13.66)
Citations	975
Total authors	338
Mean co‐authors per article (SD)	4.5 (2.34)
International co‐authorships (%)	13.3
Single‐authored publications	6

**TABLE 2:** Top 10 most frequent journals.

Journal name	*n* (%)	Quartile^a^	IF (2024)^b^
*Journal of Foot & Ankle Research*	37 (35)	Q2 (orthopaedics and sports medicine)	2.2
*Australian Health Review*	7 (7)	Q2 (health policy)	1.4
*Journal of American Podiatric Medical Association*	7 (7)	Q3 (podiatry)	0.6
*Australian Journal of Rural Health*	6 (6)	Q1 (family practice)	2.1
*BMC Medical Education*	6 (6)	Q1 (education)	3.2
*Journal of Interprofessional Care*	6 (6)	Q2 (medicine)	2.6
*PLoS ONE*	6 (6)	Q1 (multidisciplinary)	2.6
*BMC Health Services Research*	3 (3)	Q1 (health policy)	3.0
*Rural and Remote Health*	3 (3)	Q1 (emergency medical services)	2.5
*International Journal for Quality in Healthcare*	2 (2)	Q2 (health policy)	2.2

^a^ Quartile (*Source:* SCImago journal and country rank).

^b^ IF, impact factor (*Source:* Web of Science).

### 2.6 Authors, Institutions and Countries

Table 3 displays the top 10 most frequent authors of the articles, with the most published author in workforce and education being Cylie Williams who published 21 articles. Table 4 displays the top 10 most frequent institutional affiliations, with the University of South Australia leading with 85 articles.

**TABLE 3:** Top 10 most frequent authors.

Author	Articles (*N* = (%))
C. M. Williams	21 (20)
H. B. Menz	12 (11)
R. S. Causby	11 (10)
T. Haines	10 (10)
H. A. Banwell	9 (9)
S. E. Munteanu	7 (7)
A. G. Couch	7 (7)
V. Chuter	6 (6)
K. Graham	5 (5)
F. Hawke	5 (5)

**TABLE 4:** Top 10 most frequent affiliations.

Institution	Number of articles (*N* = (%))
University of South Australia	85 (81.0)
Monash University	55 (52.3)
La Trobe University	52 (49.5)
Queensland University of Technology	24 (22.9)
University of Newcastle	23 (21.9)
Western Sydney University	18 (17.1)
Charles Sturt University	14 (13.3)
James Cook University	8 (7.6)
Curtin University	7 (6.6)
University of Southampton	7 (6.6)

### 2.7 Citations

Table 5 presents the top 10 most highly cited articles according to total citations and citations per year. Articles with the highest total citations related to working in rural remote areas, footwear awareness amongst older adults, and weight bias amongst healthcare workers.

**TABLE 5:** Top 10 cited articles (total citations and average citations per article per year).

Authors (year)	Publication title	Journal	Citations (*n*)	Average citations per year
Chisholm (2011)	Measuring rural allied health workforce turnover and retention: what are the patterns, determinants and costs?	*Australian Journal of Rural Health*	69	4.60
Munro (1998)	Foot‐care awareness. A survey of persons aged 65 years and older	*Journal of American Podiatric Medical Association*	59	2.11
Lawrence (2021)	Weight bias amongst healthcare professionals	*Obesity*	49	9.80
Williams (2015)	Research capacity and culture of the Victorian public health allied health workforce is influenced by key research support staff allocation	*Australian Health Review*	47	4.27
Taylor (2017)	The process and challenges of obtaining and sustaining clinical placements for nursing and allied health students	*Journal of Clinical Nursing*	45	5.00
Smith (2009)	Allied health graduate program—supporting the transition from student to professional in an interdisciplinary program	*Journal of Interprofessional Care*	41	2.16
Snowdon (2020)	Effective clinical supervision of allied health professionals	*BMC Health Services Research*	40	5.71
Chan (2010)	Finding common ground? Evaluating an intervention to improve teamwork among primary health‐care professionals	*International Journal for Quality in Healthcare*	36	2.25
Spiers (2015)	Challenges to student transition in allied health undergraduate education in the Australian rural and remote context: a synthesis of barriers and enablers	*Rural and Remote Health*	35	3.18
Barrett (2009)	Challenges faced in implementation of a telehealth enabled chronic wound care system	*Rural and Remote Health*	29	1.71

### 2.8 Research Types and Level of Evidence

Using the UKCRC criteria, 60 articles (57%) were focused on health and social care services research, 41 (39%) on underpinning research. Other areas include the evaluation of treatments and therapeutic interventions (*n* = 2), the prevention of disease and conditions and promotion of well‐being (*n* = 1), and aetiology (*n* = 1). According to the NHMRC levels of evidence, five articles (5%) provided level I evidence, five (5%) provided level II evidence, 10 (10%) provided level III evidence, and 85 (81%) provided level IV evidence.

### 2.9 Sources of Funding

Fifty‐six articles (53%) reported no research funding. Of the remaining articles, the most common source of funding reported was from Category 3—industry and other research and development income (*n* = 22; 21%), followed by Category 1—Australian competitive grant research and development income (*n* = 12; 11%), Category 2—other public sector research and development income (*n* = 8; 8%) and Category 4—cooperative research centre research and development income (*n* = 7; 7%).

There were four overarching themes that the articles aligned with. These were:

Theme 1 Podiatry clinical practice and service delivery including access and equity of care. This included research about the diagnosis of foot and gait problems, treatment preferences, prescribing scheduled medicines, imaging, orthoses, wound care, triage, foot care provision for people with diabetes and podiatry service delivery for people who experience insecure housing.

Theme 2 Education, training, and student learning. This included research about pre‐registration podiatry teaching such as scalpel skills, simulation, eLearning, anatomy learning and peer examination. It also included evidence‐based practice training, curriculum development, competency assessment, and student transition.

Theme 3 Workforce, careers, and professional wellbeing. This included research about the podiatry profession's workforce distribution, rural retention, turnover, financial security, graduate employment, burnout, occupational stress, injuries, supervision, and career motivations.

Theme 4 Interprofessional practice and models of care. This included research where podiatry was embedded into teams of health professionals, shadowing, mixed‐discipline clinics, transprofessional roles, collaborative care, person‐centred care, and allied health primary contact models.

**FIGURE 1:**
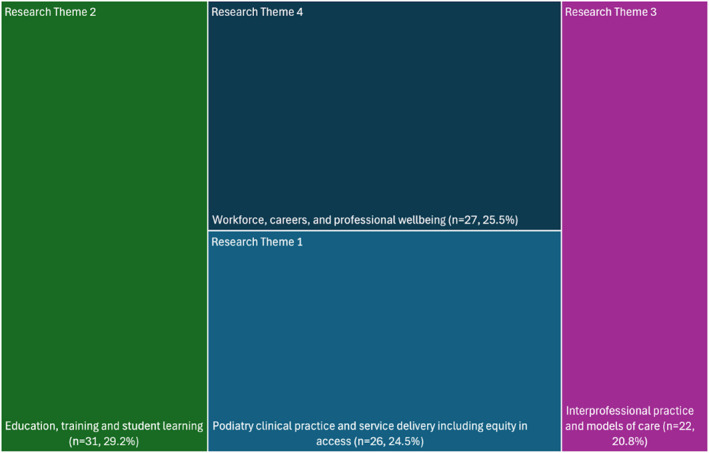
Treemap of themes of different publications.

Figure 1 displays a similar distribution of research across the four broad themes.

## 3 Discussion

Our study found that key research activity in workforce and education is driven by academics with vested interest in workforce and education issues, with little or no funding. This area has limited alignment with national health research funding schemes [19]. There were, on average, five authors per paper with 13% international co‐authorship, which speaks to the collaborative nature of research in this area. The research topics fell into four themes, highlighting research gaps within each theme.

Most research was conducted within ‘Education, training and student learning’ (Theme 2). Podiatry in Australia is taught across nine universities and education research is most commonly internally supported without external funding. This research appeared to be undertaken as part of further studies of podiatry academics in post‐doctoral or fellowship training based on the titles of the publications and funding references to research stipends or scholarships. There is little research into continuous professional development (CPD) post‐graduation. Given that CPD is a mandatory requirement of professional registration, there seems to be a gap in research into the quality and characteristics of CPD for podiatrists.

Regardless of research scheme prioritisation, allied health workforce research is widely acknowledged as a policy and planning priority [20], supported by this research (Theme 4), which accounts for the second highest number of publications. However, it is consistently challenged by the complexities of the Australian health system and limitations in data availability [21]. The fragmented nature of health services and the divided responsibility for health, particularly for the workforce, and funding between Commonwealth and state governments create a difficult environment for comprehensive health workforce research and planning [22]. Data collection for workforce research typically relies on surveys conducted during professional registration or re‐registration processes, from which annual reports are generated and made publicly available. These datasets are often maintained by government entities to inform public health priorities and enable workforce planning funded through public programs, with limited insight into the private sector. However, private industry data, such as podiatry, where the majority of practitioners work in private practice, is often sparse or entirely absent. Consequently, critical information about their workload, career intentions, business pressures, and professional motivations remains largely invisible, identifying another research gap.

**FIGURE 2:**
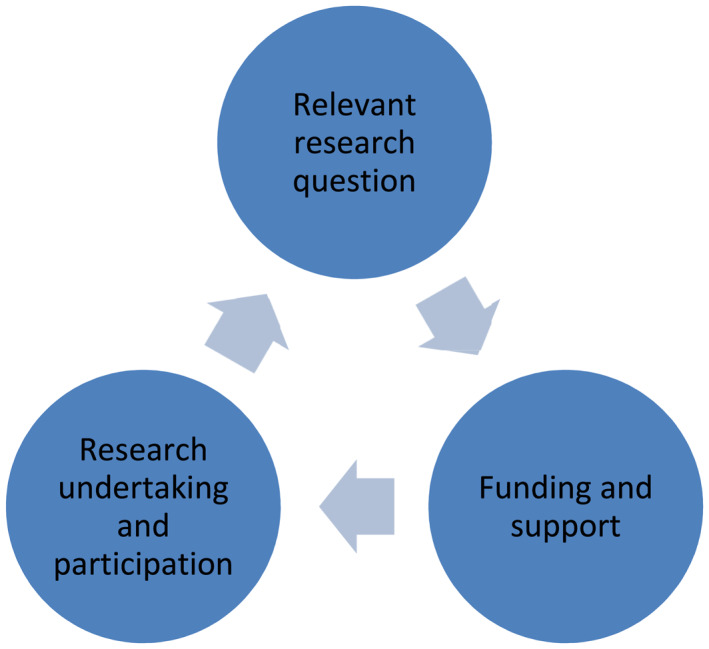
The research cycle of adequate funding to encourage research activities, which will ultimately lead to further research, thereby attracting more funding to establish a research culture within the profession.

Funding for research specific to small professions, like podiatry, is especially limited within Australia's competitive research funding landscape. These projects often fall outside national research priorities, leaving most initiatives reliant on peak professional bodies such as the Australian Podiatry Association, supported through small grants from the Australian Podiatry Education and Research Foundation (APERF), or embedded within postgraduate research. This may explain why research in clinical practice and service delivery (Theme 1) ranked third‐highest in our analysis. This underfunding creates a cyclical challenge: limited funding leads to low engagement, which then diminishes the potential to establish relevant research questions to establish a research culture within the profession to attract further funding as illustrated in Figure 2.

Interprofessional research (Theme 3) had the lowest research volume. This could be due to the relatively small profession size compared to other allied health professions, such as medicine and nursing. For example, while state governments often fund workforce surveys, for example the South Australian Allied Health Workforce survey [23], these surveys are directed at the whole of allied health research based on workforce priority areas such as research capacity [24, 25]. Podiatry's relatively small student and professional numbers may be overlooked in large studies of care workforce models undertaken primarily in publicly funded health services. This highlights the need for further support for podiatry specific workforce research not covered by large allied health focussed studies.

In the future, research must not only address recruitment and retention but also explore evolving models of care, occupational health and safety, and the integration of new technologies. Podiatrists, individually and collectively, must consider their role in advancing workforce research, whether through participation, leadership, or funding. Even with proactive engagement from within the profession, government‐driven funding for podiatry‐specific workforce research is unlikely. The challenge and opportunity lie in recognising the value of this work to the profession's sustainability and growth, identifying key research questions, and taking coordinated action to address them.

This study has several limitations. First, the analysis excluded grey literature, including conference proceedings, professional magazine commentary, working papers, and case studies; as a result, it may not capture the full breadth of workforce and education research. Second, no distinction was made between pre‐registration (undergraduate) and post‐registration training programs, such as continuing professional development, Masters, and PhD programs. This may obscure differences in reporting structures, including alternative funding arrangements such as PhD research allowances or stipends. Finally, the impact of workforce and education research may be reflected in national health and university policy rather than in citation metrics alone, which could lead to an underestimation of the true influence of research in this field.

## 4 Conclusion

Our primary research findings show that workforce and education make up a small percentage of podiatry‐related research. Most are published in low‐impact journals with low citations, but there was a high proportion of original articles spread across a range of journals, demonstrating the multidisciplinary interest in workforce and education. As a profession, we must acknowledge that opportunities for research in workforce and education exist within all aspects of podiatry. However, proactive engagement is required in developing a research culture in the profession, which could start at the university level, and be carried throughout the professional clinical career of a clinician. This will drive the development of key research questions and subsequently attract research funding.

## Author Contributions

**Malia Ho:** data curation, formal analysis, writing – original draft. **Peta Tehan:** conceptualization, formal analysis, writing – review and editing. **Matthew R. Carroll:** conceptualization, formal analysis, writing – review and editing. **Cylie Williams:** data curation, formal analysis, writing – review and editing.

## Funding

This study was supported by Australian Podiatry Education and Research Foundation.

## Conflicts of Interest

Cylie Williams is an editor of the *Journal of Foot and Ankle Research*, but had no involvement in the handling of this paper, or any within this program of research.

## Supporting information


Supporting Information S1

Supporting Information S2

## Data Availability

Data is available on request to the corresponding author.
