# Supplementary material for: Australian Podiatry Research in Workforce and Education: A Bibliometric Analysis
Source: J Foot Ankle Res. 2026 Jun 2;19(2):e70168. doi: 10.1002/jfa2.70168 (PMC13239060; doi:10.1002/jfa2.70168)
Supplement: Supplementary file 1 — Supporting Information S1 [file JFA2-19-e70168-s002.docx]

| **Workforce and education** | |
| --- | --- |
| 1. Work 2. Retent* 3. Satisfact* 4. professional* 5. Employ* 6. Develop* 7. supervis* 8. teach* 9. Learn* 10. Student 11. educat* 12. Pod* |  |
| **Search Strategy**  1: 1 or 2 or 3 or 4 or 5 or 6 or 7 or 8 or 9 or 10  2: 11  3: 1 AND 2 |  |
| **Search Restrictions** | |
|  |  |
| Year | 1970-2024 |
| Language | English |
| Source | Article |
| Author Affiliation | Australian |
